# Cold Pressor Test in Primary Hypertension: A Cross-Sectional Study

**DOI:** 10.3389/fcvm.2022.860322

**Published:** 2022-04-25

**Authors:** Yue Han, Jun Du, Jing Wang, Bin Liu, Yu-Ling Yan, Song-Bai Deng, Ya Zou, Xiao-Dong Jing, Jian-Lin Du, Ya-Jie Liu, Qiang She

**Affiliations:** Department of Cardiology, The Second Affiliated Hospital of Chongqing Medical University, Chongqing, China

**Keywords:** cold pressor test, hypertension, diuretic, heart failure, prevalence

## Abstract

**Objectives:**

To investigate the characteristics of patients with primary hypertension who had positive responses to the cold pressor test (CPT).

**Methods:**

This cross-sectional study was conducted between November 2018 to November 2019, and the CPT was performed in patients with primary hypertension in 48 hospitals. The demographic characteristics and complications were collected through a questionnaire and physical examinations. A 12-month follow-up was conducted to identify the occurrence of the following events: a) all-cause mortality; b) myocardial infarction; c) stroke; d) hospitalized for heart failure.

**Results:**

The CPT was positive in 30.7% of the patients. Compared with the negative CPT group, the positive CPT group was associated with a lower rate of blood pressure control, and was more likely to have a high salt diet, diabetes, hyperuricemia, left ventricular wall thickening, carotid plaques, coronary heart disease and heart failure. A high-salt diet (OR = 1.228, 95%CI: 1.037–1.456) was found to be correlated with the positive result of CPT. Among patients in the positive CPT group, those using diuretics had a significantly higher rate of blood pressure control than those not using diuretics (54.6 vs.42.6%, x^2^ = 6.756, *P* = 0.009). After a 12-month follow-up, the incidence of heart failure in the positive CPT group was significantly higher than that in the negative CPT group (7.35 vs.5.01%, x^2^ = 3.945, *P* = 0.047).

**Conclusions:**

Patients with positive responses to the CPT had lower rates of BP control and a high risk of heart failure, which may be related to their preference for a high-salt diet. The use of diuretics helps to better control blood pressure in those patients.

## Introduction

Hypertension is an important public health challenge worldwide and it contributes to 51% of stroke deaths and 45% of ischemic heart disease death worldwide (1–3). Studies have shown that the global prevalence of hypertension has risen continuously in recent decades, especially in low-income and middle-income countries (4). It is estimated that 26.4% (972 million) of adults had hypertension in 2000, and the number in 2025 is predicted to increase by about 60%, to a total of 1.56 billion globally (3). And hypertension is also one of the most important cardiovascular diseases in China. A national study during 2014–2017 showed that 44.7% of Chinese adults aged 35–75 years had hypertension (5). And there is a close relationship between blood pressure level and the risk of cardiovascular and cerebrovascular diseases such as stroke, atrial fibrillation, and especially heart failure. Hypertension leads to heart failure with preserved ejection fraction or heart failure with reduced ejection fraction in patients with coronary heart disease or myocardial infarction. Previous data of Chinese showed that cardiovascular and cerebrovascular diseases account for more than 40% of all deaths (6). However, the management of hypertension remains suboptimal in China, with a low treatment rate and control rate. Studies from 1997 to 2017 shows that the ranges of hypertension prevalence, awareness, treatment, and control rate among hypertensive patients were 18.0–44.7, 23.6–56.2, 14.2–48.5, and 4.2–30.1% respectively (7). Thus, it is important to improve the control rate of hypertension and prevent complications.

The cold pressor test (CPT), which was developed by a physician in the division of medicine at the Mayo Clinic Rochester, Edgar A. Hines, Jr. (1906–1978) (8), has been used as a nonspecific and strong stimulus to sympathetic neural outflow in humans. It evokes remarkable increases in blood pressure and muscle sympathetic nerve activity (MSNA) with no significant changes in heart rates (9, 10), and was used to assess the vasoconstrictor reserve (11). The reflex pathway to activate MSNA may originate from cold nociceptors in the skin that conduct afferent signals by unmyelinated C-fibers, and the pathway may involve a central vasomotor center that serves to regulate MSNA (10, 12). Therefore, CPT was also be used to evaluate the sympathetic reactivity in patients with orthostatic hypotension (13). For a long time, CPT was also used as a tool to study BP variability. Hines and Brown performed CPT in 40 healthy participants and found that the degree to which blood pressure responses to cold stimuli can be divided into three categories: a minimal increase of blood pressure in healthy participants, a much higher increase of blood pressure in hypertension, and a similar reaction to the patients with hypertension in those participants who were recognized at high risk for developing hypertension. Thus, CPT was thought to predict a subject's risk of developing hypertension. Subsequent studies have also shown that response to CPT in individuals healthy can predict hypertension (14–17). However, it is not clear whether CPT can also predict cardiovascular risk and how is the prevalence of positive CPT in patients with hypertension. Our study performed CPT in a large number of patients with primary hypertension for the first time, aim to determine the prevalence of the positive CPT in patients with primary hypertension, and to investigate the characteristics and the risk of cardiovascular events of those patients.

## Materials and Methods

### Study Design and Sample Size Estimation

This was an observational, cross-sectional study. Data from the enrolled patients were collected during the first interview and then entered into a database, including their medical and pharmacological histories. The CPT and a physical examination, including measurement of height, weight, and BP were conducted during the first interview.

**Figure 1:**
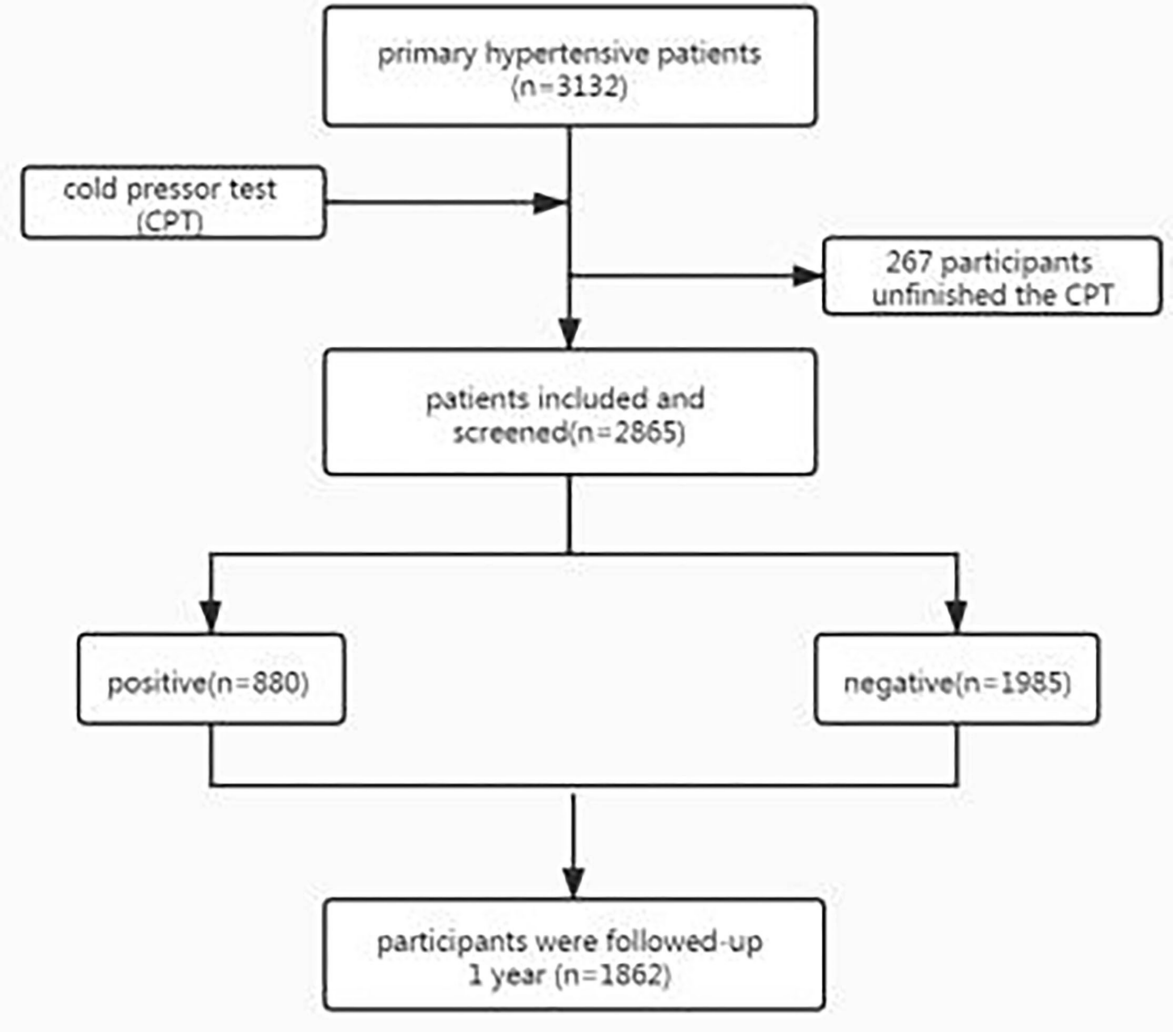
Study workflow.

A 12-month follow-up was conducted to identify the occurrence of the following events: a) all-cause mortality; b) myocardial infarction; c) stroke; d) hospitalized for heart failure (The study workflow is shown in Figure 1).

The sample size for this study was estimated based on a previous study which found that the positive rate of CPT in normotensive was32.8% (18). PASS 15.0 was used to calculate the sample size. A sample size of 1403 produces a two-sided 95% confidence interval with a width equal to 0.050 when the sample proportion is 33%.

### Inclusion/Exclusion Criteria

The CPT was performed in patients with primary hypertension in 48 hospitals from November 2018 to November 2019.

Patients were included based on the following inclusion criteria: age above 18 years and a diagnosis of primary hypertension (systolic BP ≥ 140 mmHg and/or diastolic BP ≥ 90 mmHg on at least three occasions on different days).

Patients were excluded on the basis of the following criteria: primary aldosteronism or other diseases or causes of secondary hypertension; systolic BP (SBP) ≥ 180 mmHg and/or diastolic BP (DBP) ≥ 110 mmHg; severe heart or lung diseases, acute stage stroke (<3 months) or end-tage cancer; inability to undergo the cold pressor test; or pregnancy.

### Cold Pressor Test Protocol

The participants were seated, and the blood pressure of the right upper arm was measured three times with a calibrated electronic sphygmomanometer or standard mercury sphygmomanometer after the patients had rested for 20 minutes in a room with appropriate temperature. The average blood pressure of the three measurements was taken as the basic blood pressure. Then, the participants immersed their left hands in ice water at 3–5°C for 1 minute. The blood pressure of the right upper arm was measured 0 min, 1 min, 2 min, and 4 min after removal of the left hand from the ice water. The test was considered positive if the SBP, DBP or mean arterial pressure (MAP) increased ≥ 15 mmHg after cold stimulation. The test was considered negative if the SBP, DBP or mean arterial pressure (MAP) increased <15 mmHg after cold stimulation. MAP = (SBP + 2 × DPB)/3 or MAP = DBP + 1/3 (SBP-DBP).

This study was approved by the ethical committee of Chongqing Medical University, and written informed consent was obtained from all patients participating in the study.

### Standard for Blood Pressure Under Control

The standard was based on the Guidelines for the prevention and treatment of hypertension in China (revised in 2018) (6). The target BP is <140/90 mmHg for the general population, <150/90 mmHg for those older than65 years, <140/90 mmHg for those with coronary heart disease or kidney disease (without proteinuria), and <130/80 mmHg for those with diabetes mellitus, heart failure or kidney disease (with proteinuria).

### Statistical Analysis

All data were input by two independent researchers using EpiData V.3.1 (EpiData Association, Odense, Denmark), and the database was established after checking for differences in the two records. SPSS Statistics version 21.0 (IBM, Armonk, New York) was used for statistical analysis. Categorical variables were analyzed with χ2 tests. The factors influencing CPT were screened and determined by multivariable binary logistic regression analysis. Statistical significance was defined as a *p*-value < 0.05.

## Results

### Cold Pressor Test Results

From November 2018 to November 2019, 3132 patients with primary hypertension in 48 hospitals were invited to participate in our study. The CPT was performed in 91.5% of the population (2865 patients); in the rest of the patients, it could not be performed due to cold intolerance (8.5%).

A total of 880 patients (30.7%) screened positive with the CPT and were placed in the positive group, and the other 1985 were placed in the negative group.

### Characteristics of Participants

The baseline data and complications for the included patients are shown in Table 1. The proportion of patients with a high salt diet, diabetes, hyperuricemia, left ventricular wall thickening, carotid plaques, coronary heart disease and heart failure were much greater in the positive group than the negative group (*P* < 0.05). However, there were no significant differences in the proportion of sex, age, BMI, family history of hypertension, smoking, drinking, hyperlipidemia, or higher values of serum creatinine or proteinuria between the two groups (*p* > 0.05). A high-salt diet (OR = 1.228, 95%CI: 1.037–1.456) was found to be correlated with the positive result of CPT (Table 2).

**Table 1:** Clinical characteristics of the participants.

**Characteristics**	**Total (*n*/*N*)**	**Positive group *N* (%)**	**Negative group *N* (%)**	**x^2^**	***P*-value**
**Baseline**					
Sex (male, %)	1303/2865	401 (45.6)	902 (45.4)	0.004	0.950
Age ≥ 65 years	1260/2865	397 (45.1)	863 (43.5)	0.664	0.415
Body mass index (BMI) ≥ 24 kg/m^2^	1607/2830	508 (58.7)	1099 (56.0)	1.790	0.181
Family history of hypertension	1179/2825	372 (43.2)	807 (41.1)	1.030	0.310
Current smoker	641/2865	203 (23.1)	438 (22.1)	0.353	0.552
Current drinker	579/2865	177 (20.1)	402 (20.3)	0.007	0.932
High salt diet	1496/2865	500 (56.8)	996 (50.2)	10.780	0.001
Hyperlipidemia	1015/2782	328 (38.5)	687 (35.6)	2.148	0.143
Diabetes	512/2818	179 (20.6)	333 (17.1)	4.812	0.028
Hyperuricemia	317/2700	116 (13.9)	201 (10.8)	5.625	0.018
**Complications**					
Left ventricular wall thickening	326/2649	129 (15.8)	197 (10.8)	13.033	0.000
Carotid plaques	404/2574	162 (20.5)	242 (13.6)	19.938	0.000
Higher values of serum creatinine or proteinuria	155/2620	52 (6.4)	103 (5.7)	0.567	0.452
Coronary heart disease	465/2740	168 (19.9)	297 (15.7)	7.454	0.006
Heart failure	92/2722	38 (4.5)	54 (2.9)	4.906	0.027

*Body mass index (BMI) referred to weight in kilograms (kg) divided by height in meters squared (m2). Current smoker referred to smoking at least 1 cigarette every day. Current drinker referred to drinking alcohol at least 1 time/month in the previous 1 year. A high salt diet referred to the intake of at least 6g of salt every day. Hyperlipidemia referred to either TG ≥ 2.26 mmol/L, TC ≥ 5.2 mmol/L, LDL-C ≥ 3.4 mmol/L or self-reported use of lipid-lowering drugs. Diabetes referred to the use of hypoglycemic medication or a measured fasting blood glucose level ≥ 7.0 mmol/L or non-fasting blood glucose level ≥ 11.1 mmol/L. Hyperuricemia referred to blood uric acid > 420umol/L or 7mg/dL in two tests on different days*.

**Table 2:** Analysis of factors associated with positive results of CPT.

	**B**	**S.E**.	***P*-value**	**OR (95%CI)**
Sex	0.031	0.097	0.748	1.032 (0.853–1.249)
Age	0.125	0.088	0.156	1.133 (0.953–1.346)
BMI	0.086	0.088	0.329	1.089 (0.918–1.293)
Family history of hypertension	0.078	0.088	0.374	1.082 (0.910–1.285)
High salt diet	0.206	0.087	0.018	1.228 (1.037–1.456)
Current drinker	0.077	0.123	0.528	1.080 (0.850–1.374)
Hyperlipidemia	0.047	0.273	0.601	1.049 (0.878–1.253)
Diabetes	0.186	2.952	0.086	1.205 (0.974–1.490)
Hyperuricemia	0.238	3.351	0.067	1.269 (0.983–1.637)

### Medication and Adverse Events of Participants

**Figure 2:**
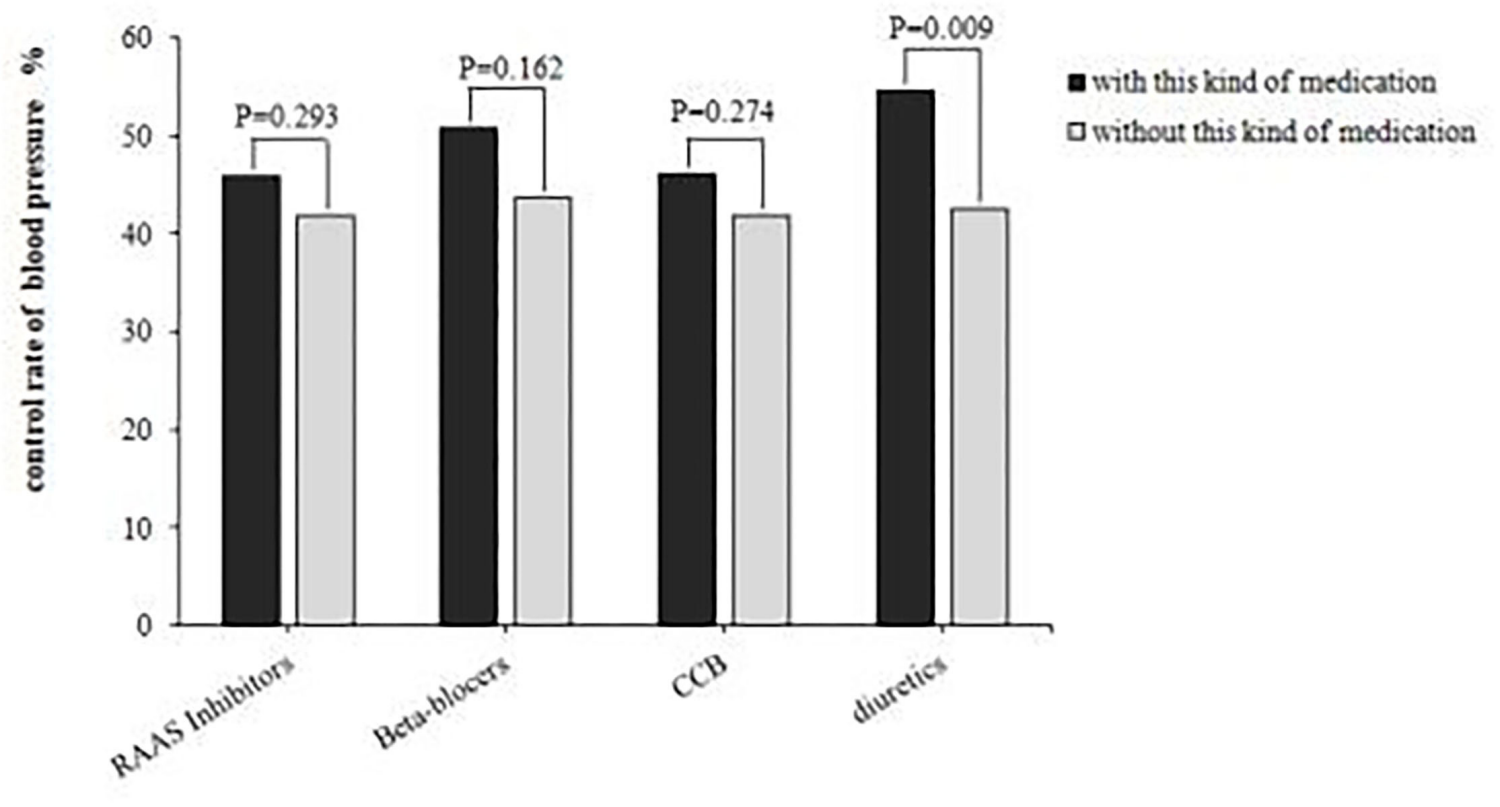
Comparison of blood pressure control rates among different medication. RAAS, renin-angiotensin-aldosterone system; CCB, calcium channel blockers.

Among the 2865 patients included in this study, 2375 patients regularly took antihypertensive drugs, including 766 (87.0%) in the positive CPT group and 1609 (81.1%) in the negative CPT group. Among the patients regularly taking antihypertensive drugs, the blood pressure compliance rate was significantly lower in the positive group than the negative group (44.8 vs.49.8%, x^2^ = 5.335, *P* = 0.021). In the positive CPT group, most patients took renin-angiotensin-aldosterone system (RAAS) inhibitors (60.5%) to control blood pressure, followed by calcium channel blockers (CCB, 58.9%), diuretics (16%), and beta-blockers (12.5%). The comparison of the blood pressure control rates among four antihypertensive therapies is shown in Figure 2. The blood pressure control rate for patients using diuretics was significantly higher than that for patients who did not use diuretics (54.6 vs.42.6%, x^2^ = 6.756, *P* = 0.009). However, there was no significant difference in whether CCB, beta-blockers, or RAAS inhibitors were used.

1,862 patients were followed up for 12-month to determine the incidence of adverse events. There was no significant difference in all-cause mortality, myocardial infarction, or stroke between the positive group and the negative group, but the hospitalization due to heart failure in the positive CPT group was significantly higher than that in the negative CPT group (Table 3).

**Table 3:** Incidence of adverse events.

**Adverse events**	**Positive group (*N* = 544) (*n*, %)**	**Negative group (*N* = 1318) (*n*, %)**	***P*-value**
All-cause mortality	2 (0.37)	2 (0.15)	0.36
Myocardial infarction	0 (0.00)	2 (0.15)	0.363
Stroke	4 (0.74)	8 (0.61)	0.753
Hospitalized for heart failure	40 (7.35)	66 (5.01)	0.047

## Discussion

CPT, which has long been a standard test for sympathetic function, has been documented to predict the subsequent risk of hypertension in normotensive persons (14–17). However, the physiological basis of the BP responses to the CPT is not yet fully understood. Studies have shown that the reason for CPT increasing BP may be the significant constriction of arterioles by inducing systemic or local sympathetic nerve activation (9, 19–22), and CPT can increase plasma norepinephrine (19, 21) and myosympathetic nerve activity (9, 20, 22). The stimulus itself is also complex and dynamic, and may cause pain and distress, which, in turn, can contribute to sympathetic activation.

Keller-Ross et al. conducted a study showing an age-and sex-dependent increase in muscle sympathetic nerve activity incidence and frequency with the CPT (23), which contributed to greater support of BP during the CPT in the older women. While, our study shows that in hypertensive patients, the BP responses to the CPT were independent of gender and age, and the patients with diabetes, carotid artery plaque, and coronary heart disease in the positive CPT group were significantly higher than those in the negative group. This may be related to vascular endothelial dysfunction in these patients. Studies have shown that healthy endothelial cells maintain low levels of oxidative stress and relax vascular tension by releasing vascular active mediators such as nitric oxide, prostacyclin I2 (PGI2), bradykinin, endothelin-1 (ET-1), and angiotensin II (Ang-II) (24–29). Imbalance of these factors can lead to endothelial dysfunction, which ultimately leads to cardiovascular complications such as arterial plaque and coronary heart disease (30, 31). Nabel et al. (32) showed that normal arteries dilated and diseased arteries contracted after CPT. In normal blood vessels, CPT may induce coronary dilation by acting on β 2-adrenergic receptors on coronary vascular smooth muscle cells via catecholamines and by stimulating α 2-adrenergic receptors on coronary endothelial cells to stimulate nitric oxide synthesis. Vasoconstriction was observed in patients with cardiovascular disease, such as coronary heart disease. This may also be the reason why coronary heart disease complications were more easily observed in the CPT positive group in our study.

Previous studies showed that BP responses to CPT vary from individual to individual in healthy people, and our study showed that in the population with primary hypertension, different individuals still had different responses to CPT, and the high response rate of CPT was 30.7%. We found that patients with CPT responsiveness were characterized by a high-salt diet. Jing Chen et al. (33) showed that BP response to the CPT was associated with salt sensitivity and potassiumsensitivity. And a low-sodium or high-potassium diet might be more effective to lower BP among individuals with high responses to the CPT, while a high sodium diet could increase systolic blood pressure in patients with positive results of CPT. These results indicate that high responses to the CPT may be related to salt sensitivity of BP.

Previous studies have also suggested that sympathetic nervous system activity might play an important role in determining the salt-sensitivity of BP (34), which affects renal hemodynamics, sodium and water processing (35, 36). Salt-sensitive hypertension is characterized by a high blood pressure response to sodium intake, which is often manifested by abnormal sodium metabolism, renal sodium retention, and enhanced sympathetic nerve activity. These pathophysiological features further lead to the retention of water and sodium. Diuretics help the kidneys get rid of excess water and sodium. Therefore, diuretics have an obvious antihypertensive effect on salt-sensitive hypertension. Our study found that patients with high responses to CPT and patients with salt sensitivity had similar characteristics, and the use of diuretics improve the control rate of BP in the positive group of CPT, which also suggested that patients with high responses to CPT may also have salt sensitivity of BP. And we also found that the incidence of heart failure was significantly higher in the positive group than in the negative group of CPT. We thought that may be related to the higher incidence of water and sodium retention in the positive group. These findings also support that patients with high responses to CPT have similar characteristics to patients with salt-sensitive hypertension. Therefore, CPT may become a new diagnostic method for salt-sensitive hypertension. However, there are still few studies on the application of CPT in the diagnosis of salt-sensitive hypertension, and we need more studies to compare CPT and acute venous saline load or chronic dietary salt load test to understand the role of CPT in the diagnosis of salt-sensitive hypertension.

## Conclusions

Patients with high responses to CPT had lower rates of BP control and a high risk of heart failure, which may be related to their preference for a high-salt diet. The use of diuretics helps to better control blood pressure in primary hypertensive patients with positive results of CPT.

## Data Availability Statement

The raw data supporting the conclusions of this article will be made available by the authors, without undue reservation.

## Ethics Statement

The studies involving human participants were reviewed and approved by the Ethical Committee of Chongqing Medical University. The patients/participants provided their written informed consent to participate in this study.

## Author Contributions

YH: methodology, performing the experiments, data curation, formal analysis, writing—original draft preparation, and writing—review and editing. JD: methodology, performing the experiments, data curation, and formal analysis. JW: performing the experiments, and data curation. BL: writing—review and editing. S-BD: validation. Y-LY: writing—review and editing. YZ: performing the experiments. X-DJ: data curation. J-LD: writing—review and editing. Y-JL: data curation. CPT Study Group: performing the experiments. QS: supervision, project administration, and writing—review and editing. All authors contributed to the article and approved the submitted version.

## Funding

This work was supported by grants from the foundation for innovative Research Groups of National Natural Science Foundation of China (81770251) and Chongqing Science and Technology Foundation (cstc2017shmsA130086).

## Conflict of Interest

The authors declare that the research was conducted in the absence of any commercial or financial relationships that could be construed as a potential conflict of interest.

## Publisher's Note

All claims expressed in this article are solely those of the authors and do not necessarily represent those of their affiliated organizations, or those of the publisher, the editors and the reviewers. Any product that may be evaluated in this article, or claim that may be made by its manufacturer, is not guaranteed or endorsed by the publisher.
